# One Health: An Effective and Ethical Approach to Leptospirosis Control in Australia

**DOI:** 10.3390/tropicalmed7110389

**Published:** 2022-11-21

**Authors:** Hong Tham Pham, Minh-Hoang Tran

**Affiliations:** 1 Department of Pharmacy, Nguyen Tat Thanh University, Ho Chi Minh City 72820, Vietnam; 2 Department of Pharmacy, Nhan Dan Gia Dinh Hospital, Ho Chi Minh City 72316, Vietnam; 3 Sydney School of Public Health, The University of Sydney, Sydney, NSW 2006, Australia

**Keywords:** leptospirosis, One Health, effectiveness, ethics, Australia

## Abstract

The increasing concerns over emerging infectious diseases and potential pandemics led to the formation of One Health, a collaborative, multidisciplinary approach to address the risks from human–animal–ecosystem interactions. This multi-sectoral approach is specifically important in Australia, a biodiverse country with unique flora, fauna, and many infectious diseases, including leptospirosis. Leptospirosis is a relatively rare but potentially fatal zoonosis, with an attributed mortality of around 60,000 deaths per year worldwide. In recent years, sporadic cases and alarming outbreaks of leptospirosis have been notified in many states and territories of Australia, noteworthily in 2018 and 2019. The sudden outbreaks in these two years have raised a question about the possibility of a more severe menace or a potential threat to both humans and animals. Amid the fight against leptospirosis, One Health has been shown to be an excellent and ideal framework, especially in Australia, the country that has taken the lead in zoonosis control using this approach. In this review, the focus will be put on the effectiveness and ethics of One Health in leptospirosis control under the Australian setting to further advocate the implementation of this framework for many other infectious diseases.

## 1. Introduction

Recent decades have witnessed increasing concerns over the risk of emerging infectious diseases and potential epidemics or pandemics [1]. The majority of these diseases are zoonoses [1,2], originating mainly from “human activities, including changes in ecosystems and land use, intensification of agriculture, urbanisation, and international travel and trade [2]”. This requires a multidisciplinary approach to respond to and manage the threats from any deadly pathogens. One Health, deriving from these concerns, is a collaborative, multidisciplinary, and cross-sectoral approach to address the risks from human–animal–ecosystem interactions [1,2,3]. One Health is specifically important in Australia, a biodiverse country with unique flora and fauna [4], which seems favourable to many communicable diseases if not properly controlled.

Leptospirosis, a contagious disease caused by the pathogenic *Leptospira* species [5], is a relatively rare but potentially fatal zoonosis [6,7], with an attributed mortality of 60,000 deaths per year worldwide [8]. While more than 250 serovars of 17 pathogenic species have been discovered [9,10], only a few are known to be the predominant pathogens in Australia, including Hardjo, Pomona, and Zanoni serovars in cattle and Pomona, Tarassovi, and Bratislava serovars in pigs [5]. The symptoms of leptospirosis can differ among animals and humans, as chronically infected animals often remain asymptomatic, whereas the manifestations in humans may lead to severe conditions, i.e., pulmonary haemorrhage, and even death [5].

In recent years, sporadic cases and outbreaks of leptospirosis have been reported in many regions of Australia [9]. The largest outbreak in humans recorded in Australia was in 2018 at a berry farm in New South Wales, when 84 cases were identified after exposure to leptospires excreted by infected mice [9]. Later, in 2019, unprecedented fatal canine leptospirosis occurred in Sydney and Melbourne, raising a question about the association between these human and dog outbreaks, which consequently led to a bigger concern as to whether these signalled a more serious threat to this South Pacific nation [9]. Most recently, in 2021, 14 cases with prior exposure to cattle were detected in an outbreak during the wet season [11], showing the on-going threat to Australia.

In this battle against leptospirosis, One Health is a potential approach [12], showing the significant prospect of limiting these contagions. By forming good relationships at governmental levels between health, human, and animal disease bodies, as well as maintaining multidisciplinary well-working communities [13], Australia has been deemed as an advocating leader for One Health in zoonosis control [9], including leptospirosis. Following on from this, One Health is a leading framework in Australia, connecting experts from different backgrounds [1,14] to implement their knowledge and experience to control the spread of the pathogen as well as to treat infected patients while still ensuring equity for every individual [15]. In this review, the effectiveness and ethics of One Health will be further discussed under the Australian setting to provide a more specific view of leptospirosis control and advocate this approach to other infectious diseases.

## 2. Epidemiology of Leptospirosis

**Figure 1:**
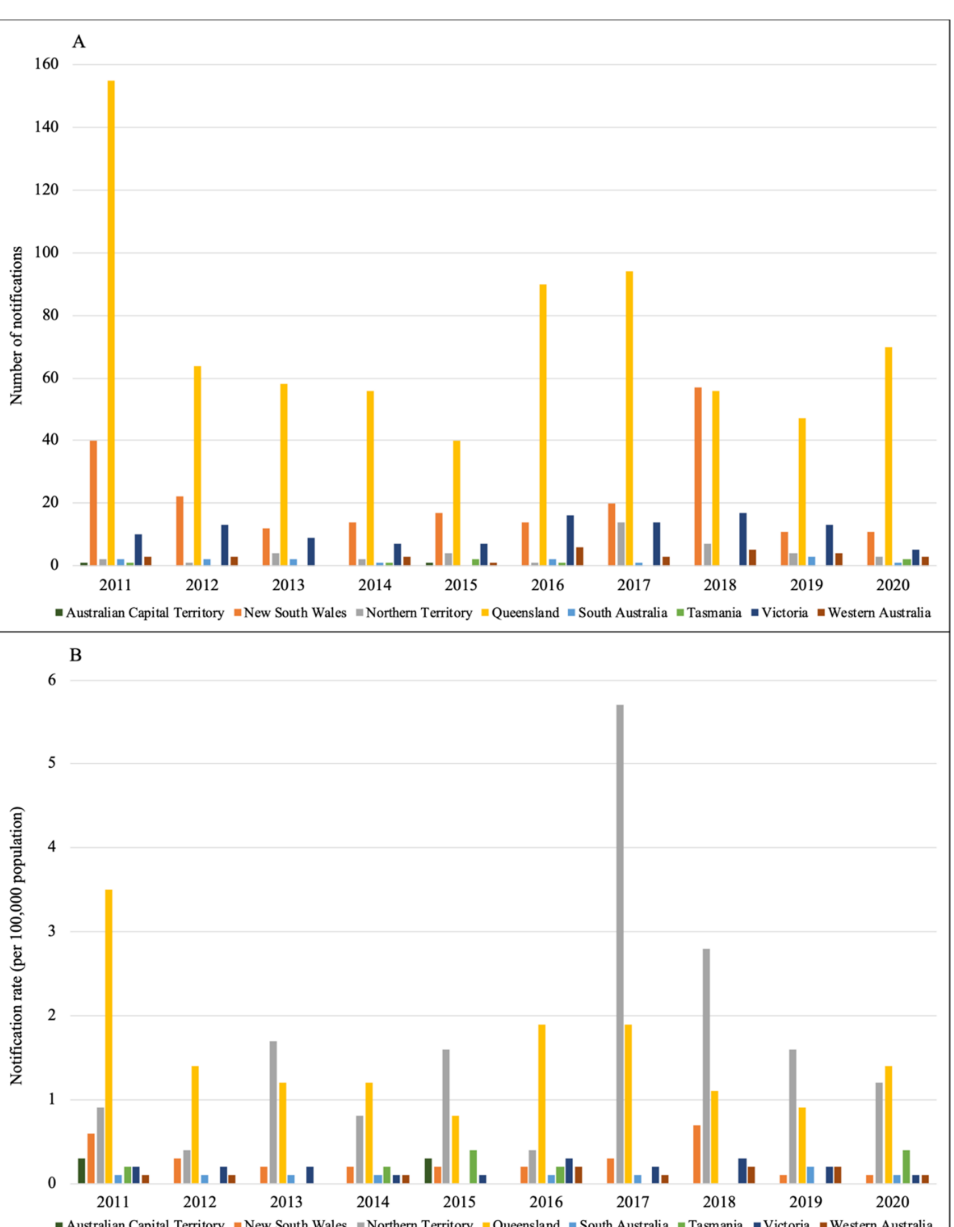
Incidence of leptospirosis notified to the NNDSS by Australian states and territories from 2011 to 2020. (**A**) number of notifications; (**B**) notification rate (per 100,000 population) [17].

**Figure 2:**
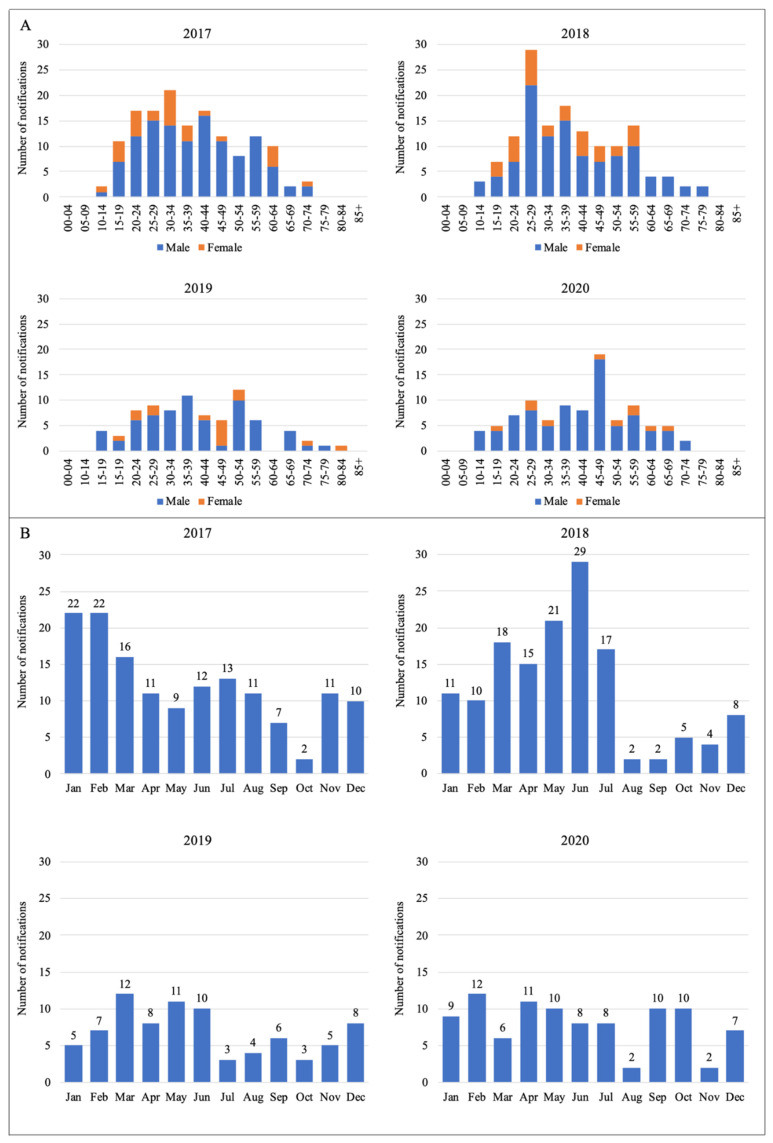
Number of leptospirosis notifications notified to the NNDSS from 2017 to 2020 by: (**A**) age and gender; (**B**) month [17].

**Distribution**. Globally, there are roughly one million severe cases related to leptospirosis per year, while this figure for Australian settings is much lower, with about 2400 cases and more than 100 deaths annually [16]. Notified data reported to the National Notifiable Disease Surveillance System (NNDSS) in Australia [17] are summarised in Figure 1 and Figure 2. In general, over the course of 10 years from 2011 to 2020, Queensland was the state with the highest number of notifications, followed by New South Wales and Victoria, respectively [17]. Within the last four years, the incidence in males was much higher than in females. Most of the cases were reported in people aged 20–60 and in the first six months of a year [17], which also coincides with the rainy season in Australia [18].

**Pathology and risk factors**. The leptospiral life cycle starts with shedding in the urine of the chronically infected animals, persisting in the ambient environment, acquiring a new host, and finally disseminating to the kidneys, turning them into reservoirs of pathogens [6], and also the primary sources of infection. The two most significant and important reservoirs are large herbivores and small mammals, including rodents, bats, etc. [6]. In terms of pathogen transmission, *Leptospira* species can enter the human body through skin cuts, abrasions, or mucous membranes in the mouth, nose, or eyes [6,7], either via direct contact with infected animals or indirect contact with urine, water, or soil contaminated with leptospires from the former hosts [6,19]. The more common latter form of transmission can be associated with occupational, recreational, or avocational exposure [6]. People working outdoors with animals, especially in wet regions, farming areas, or flooding grounds [6,7,8], are more likely to get cuts, scratches, or abrasions, thus having a higher risk of leptospirosis. While recreational activities and competitive events, including all water sports, can create potential outbreaks [6,7,8], avocational exposure can affect millions of people in tropical regions, mainly due to insufficient sanitation, poor housing, and rat exposure [6].

## 3. Effectiveness of One Health

**Diagnosis**. The diagnostic process usually starts with the patient’s symptoms and laboratory tests [20]. In complicated conditions, the isolation of the agent from the culture of microorganisms will be the most reliable evidence for diagnosis [20,21]. However, relevant knowledge is required to recognise the isolated pathogenic bacterial species and the normal microbial flora at the sampled anatomical site [21], since it requires an orientated order to select the appropriate culture media for *Leptospira*. In some cases, when physicians cannot identify the exact pathogen, understanding the epidemiology and pathology of potential infectious agents can help orientate the diagnosis. Referencing to leptospirosis, as a relatively rare bacterial disease in Australia [7], a large number of positive cases can be misdiagnosed, which might threaten patients with severe infections. Moreover, clinical manifestations of leptospirosis can range from mild symptoms to life-threatening complications [6,7,8], making it more difficult to distinguish from other possible causes. Despite this, given the role of One Health, combined information from multidisciplinary experts, i.e., an infection epidemiologist, a microbiologist, and the main physician, can form reliable evidence for an accurate diagnosis, such as the diagnosis framework of the Public Health Laboratory Network [19]. Based on the epidemiology and pathology of leptospirosis, it is important to outline the potential pathogens by investigating environmental factors as well as the patient’s history of working and travelling. The resultant recommendations can, therefore, strengthen the reliability of laboratory testing and the isolation of microorganisms, especially when it comes to serology tests or the highly precise polymerase chain reaction test [19], which eventually facilitate the physician in diagnosing the correct pathogenic species.

**Treatment**. The treatment of acute leptospirosis in humans and animals depends mostly on antibiotics and supportive measures [6,22]. However, antimicrobial therapies are usually subject to their effectiveness and safety, as well as the availability and cost of each antibiotic [22]. Since there are different characteristics among humans and animals, the corresponding choice of antibiotic also requires multi-sectoral considerations, as in the case of One Health. Another thing that physicians usually consider is the susceptibility of the pathogen to antibiotics. Many scientists and institutions have shown concerns over the risk of antimicrobial resistance, a barrier to infection treatment and a significant threat to global health security [23]. This is also one of the focuses of One Health [2,4], as resistance can start from humans, animals, or even the environment, and spread to other species, and other countries, which can worsen the infections, as the antibiotics can no longer suppress or kill the bacteria causing the diseases. This problem might have resulted from the inappropriate use of antibiotics in humans or animals. The thought that a few doses of antibiotics can treat or cure an illness [23] is probably the main factor for this issue, as consumers are not fully aware of how serious this could be. One Health experts are now addressing this threat using a multidisciplinary approach, through communication, education, and training [23], to raise the awareness of every person about the rational use of antibiotics. While the last two actions are mainly for prescribers and dispensers across human and animal health [23], communication is vital for the implementation of One Health in Australia. A study in 2018 reported a “lack of inter-sectoral trust” between the veterinary and medical sectors [3], implying that there is a need for mutual trust and effective communication as well as collaboration. If both sides can cooperate and manage the appropriate use of antibiotics in their sectors, the risk of antimicrobial resistance is very likely to be reduced significantly. In the case of leptospirosis, although no significant risk of antimicrobial resistance has been reported [24], the appropriate use of antibiotics against leptospiral pathogens still needs to be monitored multi-sectorally. If implemented properly, One Health could support the healthcare system with a better approach in treating any infectious diseases.

**Prevention.** In general, preventing contagious diseases requires knowledge about the sources, cycle, and pathology of infections. Unlike many other infections, leptospirosis is an endemic zoonosis [6], highly prevalent in tropical regions [6,7,8]. Therefore, effective preventive approaches should also engage scientists from multiple backgrounds, including doctors, veterinarians, epidemiologists, microbiologists, and environmentalists. To prevent the infection, the first thing to do is block the entrance to the body of the pathogen, while attempts to stop the spread should focus on how it leaves the body and exists in the ambient environment. For the former task, regarding leptospirosis, One Health experts aim to mitigate exposure to leptospiral strains, mostly from animals or the environment [6,7,8]. In Australia, occupational exposure was reported to be the major risk for leptospirosis [8]. People working in tropical or rainfall regions, livestock or dairy farms, or military areas are highly recommended to wear personal protective equipment [6,7,8], which can prevent cuts, abrasions, or skin damage from daily activities. In addition, these individuals should also wash their hands after exposure to animal-urine-contaminated soil or water, especially those with open wounds. These simple but effective measures are mainly the efforts of a systematic multidisciplinary collaboration, from identifying the risk to deterring it from entering the body. 

More importantly, this One Health approach might have broadened the scope of preventive measures, from defence to control and eradication (e.g., the Communicable Diseases Network Australia [25]). Knowledge about the life cycle of leptospirosis is perhaps a “game-changer” in the battle against this infectious disease, starting with the primary hosts of leptospiral strains, small mammals and large herbivores. The diagnosis and treatment of infected domestic animals [6] are the first steps to separate the pathogen from the natural habitats. Additionally, scientists have cooperated to upgrade their strategy by implementing vaccination for animals [6,8], especially livestock, which can effectively control the spread of *Leptospira* species and has the potential to eradicate the pathogen from the human and animal community. However, practical and cost challenges can deter this strategy, especially in the Northern Territory of Australia [11]. To advocate the vaccination rollout, a rational allocation of resources should be considered, which can be made feasible by targeting the most vulnerable regions.

Furthermore, the ambient environment where the leptospiral strains temporarily exist should also be investigated. As rodents are potential reservoirs, measures targeting these mammals’ habitats, including the removal of rubbish and food sources, are advocated [7,8], particularly in rat-infested areas. Not only can this prevent the spread of leptospirosis but also other vector-borne diseases related to rats. In recent years, the interaction between urbanisation and climate change has been reported to associate with the incidence and frequency of leptospirosis outbreaks [6], suggesting the importance of other environmental factors in preventing this emerging infectious disease. This requires an interdisciplinary engagement of public health experts and environmentalists for a deeper understanding of this effect. Further One Health-based studies are also necessary to interpret this interaction and propose the solutions to address its consequent outcomes. Besides, any war has its price, and so does ours against the pathogens. Therefore, any measure being conducted must have taken into account its feasibility and adverse effects on humans, animals, and the environment. For instance, streptomycin/dihydrostreptomycin, once used for effective leptospirosis treatment, is no longer used in Australia due to safety concerns surrounding these antibiotics [5]. While this is just a simple case, most of the time it is nearly impossible to examine all aspects of the issue independently, especially when it comes to the complex system of all related living things. Hence, One Health is a comprehensive approach, allowing us to stop the invasion of fatal pathogens into the body while still minimising the stakes for the ecosystem.

## 4. Ethics of One Health

**Harm principle**. This principle can relate to those deliberately trying to harm, or neglecting their responsibilities, which might inflict harm on other people [26]. In the context of leptospirosis, an outbreak can occur despite the absence of those conditions [26], which can push authorities or governments to more extreme measures. Under certain circumstances, when situations are out of control, to prevent potential outbreaks, governments may demand the mass culling of infected animals as a result of the harm principle [27]. While this has not been the case with leptospirosis in an Australian setting because of effective and ethical prevention, these killings have been reported in some other infectious diseases [26]. However, as there can be “various kinds and amounts of harm” with different extents of priority [26], given the fact of limited resources, the process of identifying which needs to be addressed first is a challenge for governments, as well as the harm principle itself. This principle does not imply any comparison of different harms, or which ones should be prioritised [26]. However, with the One Health approach, this issue would not be so difficult. By exploring the interactions among humans, animals, and the ecosystem [2,3], all the risks and associated harms of each measure, compared to its potential benefits, could be critically analysed and evaluated [27]. After considering and weighing the two sides of these responses in an economic and social context, in an ideal way, One Health experts can propose their suggestions to the authorised agencies for further assessment, which could help to clarify the harm principle. Ultimately, rather than showing a total preference for the human community [28], the One Health approach tends to balance or harmonise the individual and collective interests of both humans and animals. This could partially explain why leptospiral-infected animals have not been culled to stop the spread of the bacteria in Australia and possibly in other nations. 

**Duty of care**. The original concept of this aspect was to clarify the responsibilities of healthcare workers in the event of fatal outbreaks [29], as this might put them at higher risks of exposure while attempting to ensure the well-being of other people. However, health is not simply the state of an individual in human society. It can only be achieved from the balance of the human–animal–environment system, as reflected in the definition of One Health [30]. Thus, the idea of duty of care needs an expansion to address all the possible related issues. In the case of leptospirosis, from a One Health perspective, efforts and resources should be allocated to all three sectors of humans, animals, and the ecosystem. Preventing contagion and treating the infected are the responsibilities of physicians and public health specialists, while veterinarians need to ensure the immunisation of animals known to be the hosts of *Leptospira* species. Measures to maintain the safety of the environment must also be carried out to mitigate the presence of the pathogen in the natural habitat, i.e., keeping livestock away from the water source in suspected areas. In regions that usually suffer from floods and leptospirosis, assistance from One Health experts to minimise the damage and consequent risks could prevent disaster after disaster. Provided that the duty of care principle aims at avoiding predictable actions that might cause harm to other people, One Health is an ideal approach, allowing us to foresee all the potential risks and the accompanying solutions.

**Solidarity.** At the most basic level, solidarity “comprises manifestations of the willingness to carry costs to assist others with whom a person recognises sameness or similarity in at least one relevant respect” [31]. This could also be understood as a united social relation of individuals, groups, or communities at a national or global scale [32]. In the healthcare setting, solidarity is “generally linked to a sense of commitment to help those in need” and “a driving force for societal organizations” [33], implying the necessity to capitalise on this resource, particularly in infectious disease control. Coincidently, the principle of solidarity and One Health appear to have one vital thing in common, making this inter-disciplinary approach the perfect tool in times of outbreaks and contagions. They both mean the unification of smaller parts into a whole, for the greater good of every aspect. Therefore, One Health can inherit the spirit of solidarity and turn it into the inspiration to fight against outbreaks. More notably, experts from multiple backgrounds may form an alliance under the name of One Health to mobilise necessary resources from communities and facilitate the redistribution of these resources [33] for different priorities transparently. Back to the case of leptospirosis, as poor housing and floodwater are important known risk factors [6], whereas the government often appears to lack interest in controlling this zoonosis [5,34], the support of the One Health “alliance” could be critically important to control infection and the spread of the bacteria. Besides, the spirit of solidarity might be expanded to animals and environments, contributing materially to the well-being of humans [35]. This implies an opportunity to resolve the lack of trust between the medical and veterinary sectors in Australia [3], and later to facilitate their unity. Therefore, people with high exposure to the leptospiral strains are more likely to benefit from this spirit of solidarity, suggesting the applicability of this principle to many other emerging infectious diseases.

**Healthcare equity**. In close association with solidarity, One Health has a significant potential to promote healthcare equity, specifically in low- and middle-income countries [15]. Following the redistribution of resources from the spirit of solidarity, people from difficult backgrounds and suffering from the disease burden may have another chance to overcome these adversities. For people in areas where addressing zoonoses is not a priority for resource allocation, or those unable to afford the treatment, One Health also comes up with another opportunity. Given the fact that many infectious diseases are preventable [15], governments may consider the One Health preventive measures, as discussed above, to control the sources of infections. Accordingly, this would lower the risk of contagion for all those affected by the disease, regardless of socioeconomic status. This approach is expected to improve well-being in regions with socioeconomic and health inequities, i.e., Africa, the continent where people are at the highest risk of endemic zoonoses [15]. Additionally, with One Health, the concept of equity can probably be extended for other animals as well. While the idea may sound absurd, the effectiveness of animal vaccination, particularly against leptospirosis [6], could be a positive sign of consensus on this matter. After all, if treating animals equitably is shown to be a significantly cost-effective measure, why not consider it? Hence, ensuring equity for all of the living is probably one of the justifications for the ethical aspects of One Health in infection control.

**Research ethics.** One Health plays an essential role in ensuring research ethics, as it is the key to maintaining all of the above ethical aspects of this approach. The foundation of One Health is based in multiple fields, from health to the social and environmental sciences [1,2], implicating a diverse background with various interests. Although there is still no agreement on One Health ethics [36], for these interventions to be successful, “they must address fundamental ethical questions about what is valuable, what is to be protected and, ultimately, what is dispensable” [37]. As long as One Health can address these ethical standards, it will continue to contribute to the sustainability of humans, animal communities, and the ecosystem. However, as “significant differences still remain between One Health approaches that prioritise human interests and those seeking to protect the interests of, and distribute benefits to, non-humans” [37], it appears that there is, to some extent, a need for a new ethical framework that can best balance the interests of all living things under One Health. In spite of this, in the present context, the ethical standards of One Health remain a typical scientific approach for studies that investigate emerging infectious diseases [38]. By following a systematic approach, results from these studies can be easily applied in many contexts or settings and are more likely to be accepted when it comes to a complex and controversial situation.

## 5. Conclusions

In conclusion, as prioritisation and resource allocation always require comprehensive considerations, including ethical principles, effectiveness, socioeconomic status, etc., a multi-sectoral approach such as that advocated by One Health is critically useful and necessary to address all of the related issues in leptospirosis control. The feasibility of One Health is mainly reflected in the context of economic, social, and political forces. Whereas One Health’s effectiveness is best expressed in the process of diagnosis–treatment–prevention, it is better to consider its necessity by analysing the underlying ethical principles. Together, both aspects can form a bigger picture about the role of the One Health approach in controlling leptospirosis as well as other infectious diseases and how to properly implement it in real settings.

## Data Availability

The supporting data were publicly available at the NNDSS website.
